# Glutathione transferases: substrates, inihibitors and pro-drugs in cancer and neurodegenerative diseases

**DOI:** 10.1038/s41389-017-0025-3

**Published:** 2018-01-24

**Authors:** Nerino Allocati, Michele Masulli, Carmine Di Ilio, Luca Federici

**Affiliations:** 1 Department of Medical, Oral and Biotechnological Sciences, University “G. d’Annunzio”, Chieti, Italy; 2 CESI-MET, University “G. d’Annunzio”, Chieti, Italy

## Abstract

Glutathione transferase classical GSH conjugation activity plays a critical role in cellular detoxification against xenobiotics and noxious compounds as well as against oxidative stress. However, this feature is also exploited by cancer cells to acquire drug resistance and improve their survival. As a result, various members of the family were found overexpressed in a number of different cancers. Moreover several GST polymorphisms, ranging from null phenotypes to point mutations, were detected in members of the family and found to correlate with the onset of neuro-degenerative diseases. In the last decades, a great deal of research aimed at clarifying the role played by GSTs in drug resistance, at developing inhibitors to counteract this activity but also at exploiting GSTs for prodrugs specific activation in cancer cells. Here we summarize some of the most important achievements reached in this lively area of research.

## Introduction

The superfamily of glutathione transferases (GSTs) is composed of multifunctional proteins widely distributed in nature, in both eukaryotes and prokaryotes^1–5^. In eukaryotes, GSTs are divided according to their cellular localization into at least three major families of proteins, namely cytosolic GSTs, mitochondrial GSTs and microsomal GSTs^1,2,6^. Cytosolic GSTs are spreadily distributed and in turn divided into several major classes on the basis of their chemical, physical and structural properties. Mitochondrial GSTs are also known as kappa class GSTs and are soluble enzymes which bear structural similarities with cytosolic GSTs. On the contrary, microsomal GSTs, also known as MAPEG (membrane-associated proteins involved in eicosanoid and glutathione metabolism), are integral membrane proteins which are not evolutionary related to the other major classes.

This review focuses on the main substrates, inhibitors and reactions played by cytosolic GSTs, with an eye on their relevance for human disease; as to mitochondrial GSTs and MAPEG, we refer the reader to other excellent reviews^7,8^.

**Fig. 1:**
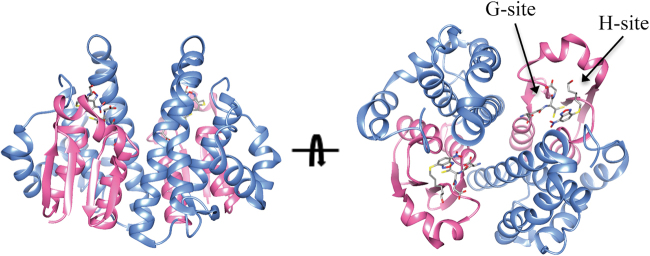
Structure of a representative GST. The structure of human GSTP1-1 in complex with GSH and the inhibitor NBDHEX is shown (pdb code 3GUS). The N-terminal thioredoxin-like domain is coloured in pink while the all-helical C-terminal domain is coloured in cyan. The G-site is occupied by a GSH molecule while the H-site is occupied by a NBDHEX molecule which are shown in sticks

Cytosolic GSTs are classified on the basis of sequence similarities and structural properties. Many classes have been recognized so far, some of them with multiple members which may share sequence identities around 40%. Interclass sequence identities are instead around 25% or less^6^. Despite the low sequence identities, all cytosolic GSTs share a common fold, which is also largely conserved in mitochondrial GSTs (Fig. 1). In humans, members of the following classes of cytosolic GSTs are present: alpha, zeta, theta, mu, pi, sigma and omega^6^. GSTs are dimeric enzymes. Usually dimers are made from identical chains but heterodimers made of two different chains from the same class are also found. Two distinct domains are recognized in each GST monomer: a N-terminal thioredoxin-like domain and a C-terminal alpha-helical domain. The first domain is responsible for GSH binding, with the presence of a specific binding task which is termed G-site. Within this site, a specific residue activates the GSH cysteinyl side chain through hydrogen-bonding. In some classes this residue is a tyrosine while in some other is a serine or a cysteine. In humans, alpha, mu, pi and sigma isoenzymes contain a tyrosine in the G-site while the other classes a serine or a cysteine. Mitochondrial kappa class enzymes, contain a serine in the G-site and resemble theta class enzymes. However they also show differences in the overall fold with the presence of a DsbA-like domain inserted within the N-terminal thioredoxin-like domain^6^. The C-terminal domain contributes, together with the N-terminal domain, to shaping the co-substrate binding site, which is termed H-site, from the hydrophobic nature of co-substrates. The great variability of GSTs co-substrates is reflected in the different H-sites shapes and chemical characters found among classes.

## GST conjugation activity

**Fig. 2:**
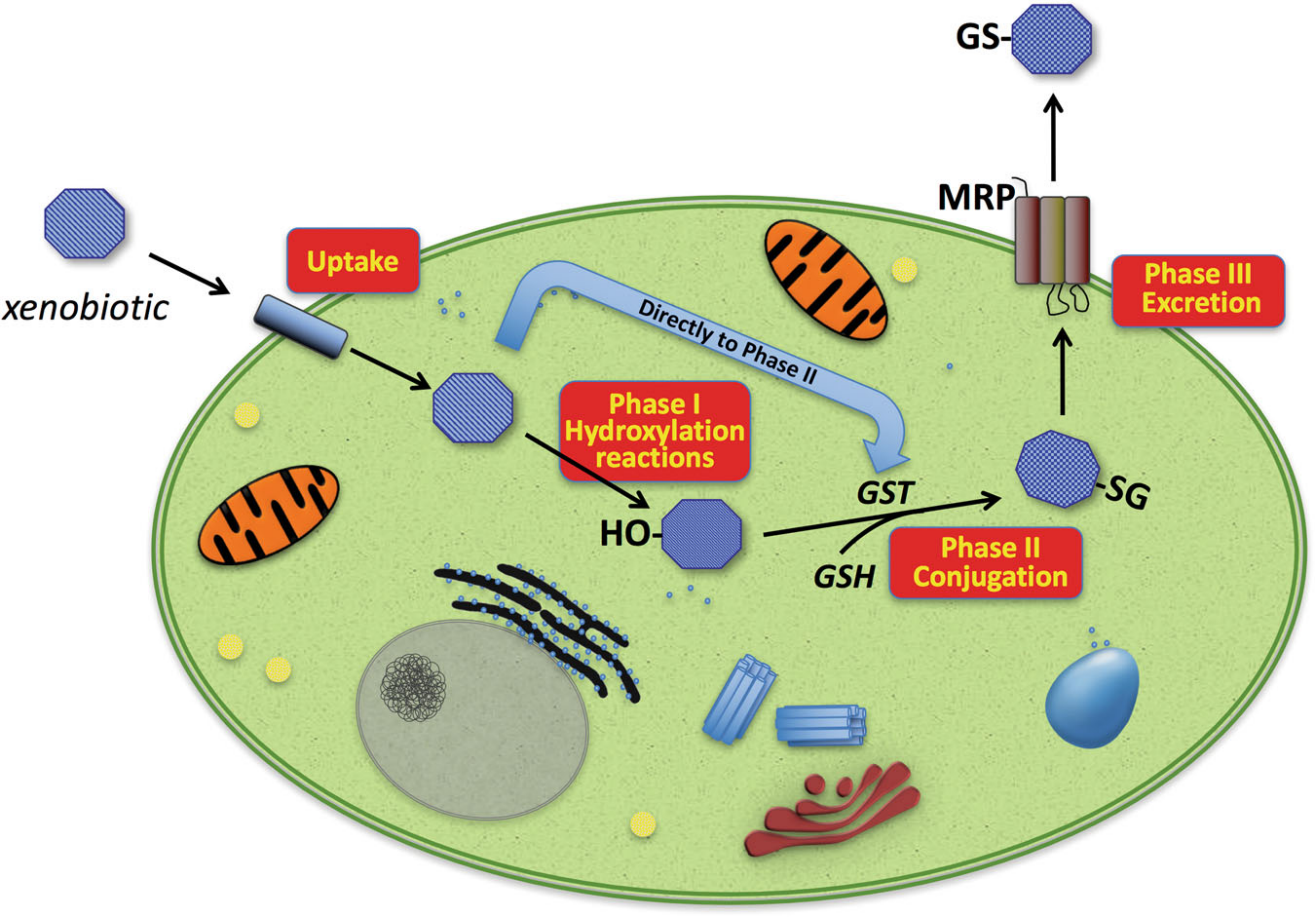
Overview of enzymatic biotransformation of xenobiotics. Harmful molecules may diffuse across the plasma-membrane and, inside cells, they may be targeted by the enzymes of the so-called Phase I metabolism. Main ones belong to the cytochrome P450 family, comprising several enzymes catalyzing different reactions including hydroxylation—the major reaction involved—oxidation and reduction. In the subsequent Phase II metabolism, the main role is played by GSTs that catalyze the conjugation of Phase I-modified xenobiotics to endogenous GSH. The conjugate obtained is then actively transported out of the cell by different transmembrane efflux pumps (Phase III). Some compounds may enter Phase II metabolism directly

GSTs have multiple biological roles, including cell protection against oxidative stress and several toxic molecules, and are involved in the synthesis and modification of leukotrienes and prostaglandins^1^. As an example, GSTs protect cellular DNA against oxidative damage that can lead to an increase of DNA mutations or induce DNA damage promoting carcinogenesis^9^. GSTs are able to conjugate glutathione (γ-l-glutamyl-l-cysteinyl-glycine, GSH) to a wide range of hydrophobic and electrophilic molecules including many carcinogens, therapeutic drugs, and many products of oxidative metabolism, making them less toxic and predisposed to further modification for discharge from the cell (Fig. 2)^1^.

A few examples of the detoxification role played by GSTs through their conjugation activity are as follows:

### Aflatoxin B1

**Fig. 3:**
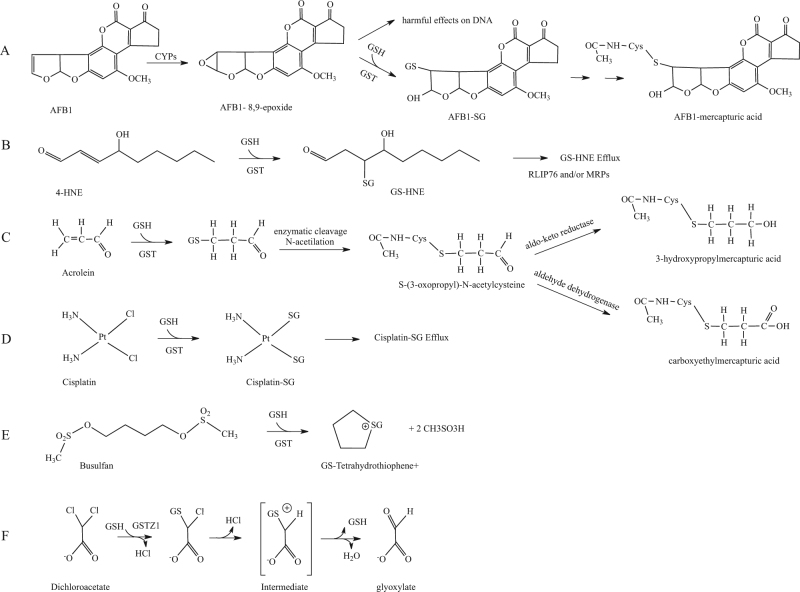
GST substrates. **a** First two steps of the metabolism of aflatoxin B1; **b** detoxification pathway of 4-hydroxynonenal involving GST; **c** GST-catalyzed conjugation of acrolein and subsequent trasnformation steps **d** enzymatic inactivation of cisplatin catalyzed by GSTs; **e** first step of the metabolic pathway of busulfan; **f** enzymatic inactivation of dichoroacetate leadind to glyoxylate

Aflatoxins are harmful metabolites produced by several *Aspergillus* species. They are highly toxic to the liver and are amongst the major identified risk factors for hepatocellular carcinoma^10–12^. Aflatoxin B1 (AFB1) is present in the environment as contaminant of cereal crops and groundnuts. The toxic effects of AFB1 arise through its enzymatic activation by cytochromes P450 to form highly reactive AFB1-8,9-epoxides. AFB1-*exo*-8,9-epoxide is then able to bind to guanine residues in DNA thus confering mutagenic properties. GSTs are involved in the main detoxification route of AFB1-*exo*-8,9-epoxide by catalyzing the conjugation of AFB1-*exo*-8,9-epoxide to endogenous GSH. The conjugation adduct obtained is then eliminated by the mercapturic acid pathway (Fig. 3a). Drugs-induced increased expression of GSH could play an important role in the protection against the toxic effects of AFB1^12^.

### 4-Hydroxynonenal

4-Hydroxynonenal (4HNE) is a lipoperoxidation-derived aldehyde that can damage proteins and DNA through the generation of covalent adducts and is implicated in the control of cell signaling^13,14^. Intracellular concentrations of 4HNE may be critical for cells^13^. High concentrations of 4HNE have been associated with different processes such as apoptosis, cell differentiation, altered gene expression and with several diseases correlated to redox imbalance such as neurodegenerative and cardiovascular diseases, metabolic syndrome, and cancer^15^. Several enzymatic pathways are involved in protecting cells against 4HNE injury. The major detoxification pathway of 4HNE involves GSTs^16^. GSTs conjugate 4HNE to GSH, and the corresponding glutathionyl-4-HNE (GS-HNE) is actively transported out of cells via RLIP76, a multifunctional membrane transporter for GSH-conjugated compounds, and/or MRPs (multidrug-resistance proteins; Fig. 3b)^15,17^.

### Acrolein

Acrolein is a highly reactive aldehyde used in the synthesis of several organic compounds in chemical industry and as a biocide in agriculture. Acrolein can be formed by burning tobacco, wood, plastic and fuels, and in the heating of animals and vegetables fats and oils at high temperatures. It is also formed naturally in small amounts in the body as an end-product of lipid oxidation and the metabolism of α-hydroxyamino acids^18^. Populations exposed to high concentrations of this toxic compound include smokers and second-hand tobacco smokers. Acrolein exerts its toxic effects through inhalation, ingestion and dermal exposure. Cardiovascular tissues are particularly sensitive to the compound^19^. Acrolein can react and form adducts with DNA, lipids and proteins leading to cellular damage in several human tissues. GST is involved in the first step of the main pathway for elimination of acrolein from the body^20^. GSTs catalyze the conjugation of acrolein to GSH and the corresponding conjugate is further modified by the enzymatic cleavage of the GSH glutamic acid and glycine residues by γ-glutamyltranspeptidase and cysteinylglycinase, respectively. The cysteine conjugate product is then N-acetylated by N-acetyl-transferase^20^. At this point further modifications catalyzed by aldehyde dehydrogenase and aldo-keto reductase allow to obtain carboxyethylmercapturic acid and 3-hydroxypropylmercapturic acid respectively, which are escreted with urine (Fig. 3c).

## GST's other enzymatic activities

In addition to the classical conjugation reactions, GSTs have a role in several other catalytic functions. GSTs exhibit glutathione peroxidase activity and catalyze the reduction of organic hydroperoxides to their corresponding alcohols. Among the compounds that the enzyme reduces there are phospholipids, fatty acids and DNA hydroperoxides produced by lipid peroxidation and oxidative damage to DNA^1^. GSTs also show thiol transferase activity and have a role in thiolysis and isomerization reactions^1^. Three different mechanisms of GST-catalyzed isomerization are known: (i) carbon-carbon double bond shifts, (ii) intramolecular redox reactions and (iii) cis-trans isomerizations^21^. As an example, GSTZ1-1 catalyzes the cis-trans isomerization of 4-maleylacetoacetate to 4-fumarylacetoacetate within the phenylalanine catabolic pathway^21^. Microsomal prostaglandin E_2_ synthase-1 (MPGES-1), a member of the MAPEG family, is implicated in the biosynthesis of prostaglandin E_2_—a metabolite of arachidonic acid—involved in several biological functions. MPGES-1 catalyzes the terminal step of prostaglandin E_2_ synthesis through a GSH-dependent isomerization reaction^22^. MPGES1 have a key role in inflammatory diseases and is high upregulated in tissues during inflammation and overexpressed in tumors^23^.

## GST non-enzymatic functions

**Fig. 4:**
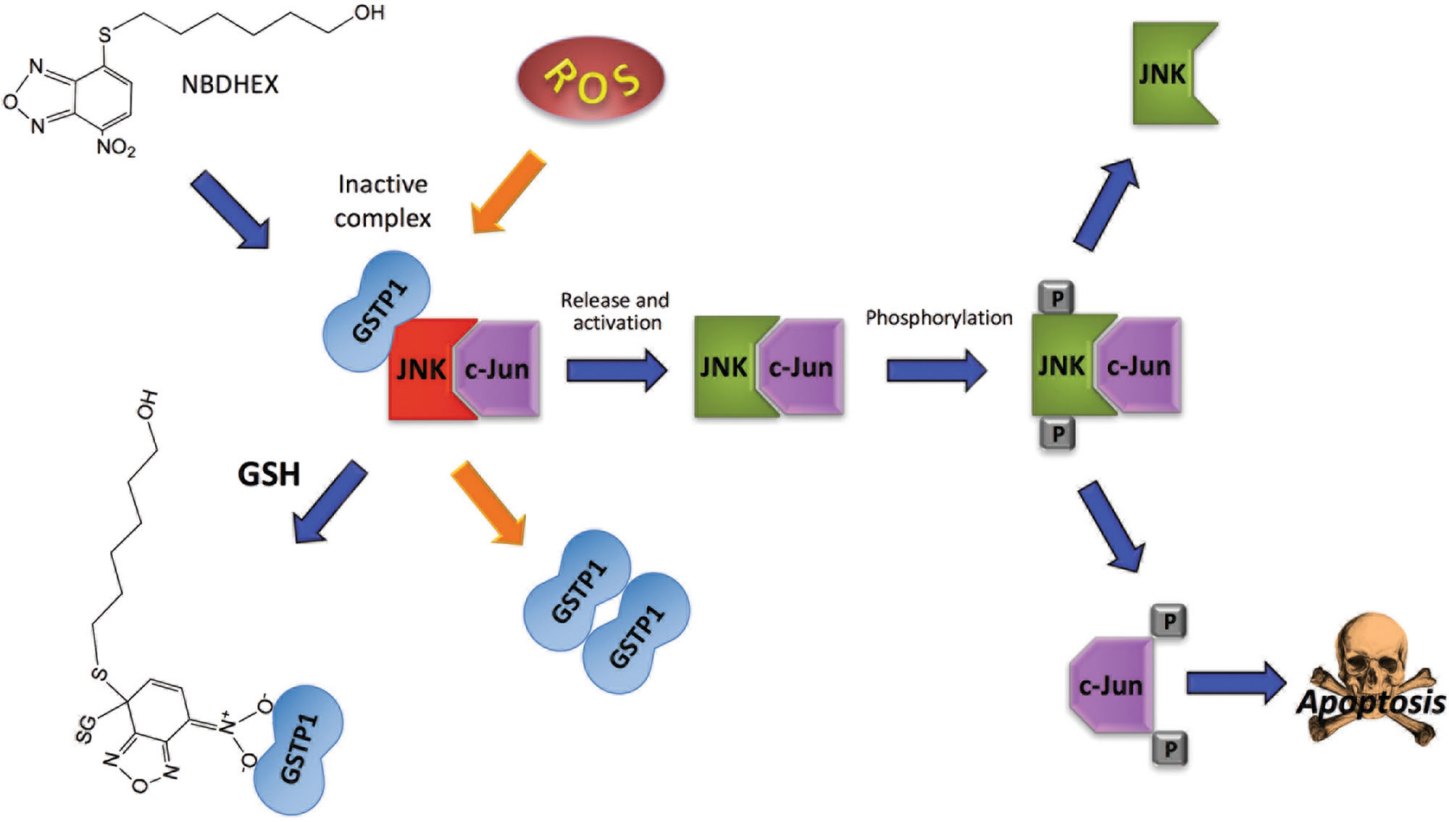
Role of GSTP1-1 in JNK signaling pathway. Monomeric GSTP1 protects tumoral cells from apoptosis by inhibition of the JNK signaling pathway through the formation of a GSTP1-JNK-cJun complex that hampers c-Jun phosphorilation. Under stress conditions, GSTP1 may dissociate from the complex and dimerize, thus enabling JNK to phosphorilate c-Jun. This event may also be triggered by the GST inhibitor NBDHEX which binds GSTP1 and induces its release from the complex

GSTs are also implicated as modulators of signal transduction pathways implicated in cell survival and apoptosis, where they control the activity of members of the mitogen-activated protein kinase (MAPK) family^24,25^. In particular, GSTP1-1 is able to protect the tumor cells from apoptosis signals by inhibiting c-Jun N-terminal kinase (JNK)—a member of the MAPK pathway—non-catalytically, by direct protein–protein association^25,26^. In response to different extracellular stimuli, the complex may dissociate and JNK can phosphorylate c-Jun, a component of the activator protein-1 transcription factor. This in turn leads to the induction of AP-1-dependent target genes, involved in cell proliferation, DNA repair, and cell death^27^. Mechanistically, it has been observed that GSTP1-1 binds, in its monomeric form, both c-Jun and JNK, thus forming an heterotrimeric complex that inhibits the phosphorylation of c-Jun by JNK. The dissociation of the enzyme from JNK also results in the dimerization of GSTP1-1 (Fig. 4)^26,28^. Furthermore, GSTP1-1 has also been shown to bind and inhibit tumor necrosis factor receptor-associated factor 2 (TRAF2), an upstream activator of JNK, thus blocking the MAPK/JNK signaling cascade at multiple levels^29^. A detailed analysis of complex formation suggested that GSTP1-1 engages TRAF2 both through its G and H sites. However, and interestingly, data also suggested that while GSTP1-1 engages TRAF2 in its dimeric form, only one monomer is involved in binding and therefore the other may still be catalitically competent^30^. Finally, a cell cycle-dependent variation of the amount of GSTP1-1-TRAF2 complex existing in cells was detected, with a maximum in G_0_/G_1_ and a strong decrease in S, G_2_, and M phases.

## GSTs gene polymorphisms and correlation to diseases

GSTs are represented—and expressed with unique patterns in each organ—in the human genome by multiple genes that are located in class-specific clusters on different chromosomes^31–33^. Several of these genes are polymorphic, resulting in phenotipic variants of GSTs. Genetic polymorphisms are heritable changes in DNA sequences as consequence of single-nucleotide polymorphisms or stable point mutations that may partially alter enzyme activity, or large deletions in coding sequences resulting in a null phenotype^24,34^. The main *gst* genes described as polymorphic are those coding for GSTM1-1, GSTT1-1 and GSTP1-1. The most common polymorphisms of GSTM1-1 and GSTT1-1 gene loci consist in the complete deletion of the genes. Furthermore, polymorphisms were also observed in other isoenzymes of the mu class: i.e. GSTM3-3 and GSTM4-4. As to the pi class, GSTP1-1 point mutations in both exon 5 (codon 105) and exon 6 (codon 114) were observed and shown to have an impact on the enzymatic activity^32^. Polymorphisms are also present in GSTs of the alpha class and omega class, with the isoenzymes GSTA1-1, GSTA2-2 and GSTO1-1 and GSTO2, respectively^32^.

Numerous studies centred on GST gene polymorphisms as factors modulating the risk of developing cancer^32^. In particular, GSTT1-1 and GSTM1-1 null genotypes, as well as GSTP1-1 variants, were studies and showed a positive correlation with cancer risk (Table 1)^24,32,35–42^. More recently, a meta-analysis study linked GSTT1-1 null polymorphism with an increased risk of coronary heart disease^43^. In addition, a significant correlation between *GSTA1-1* and *GSTT1-1* particular genotype combinations and the risk of psoriasis was observed^44^.

**Table 1:** GST allelic variants associated with cancer risk and other diseases

Gene	Allelic variant	Modification	OMIM number	Diseases	Ref.
*GSTM1*	GSTM1*O	Gene deletion	138350	Uterine leiomyoma, hypertension, oral leukoplakia, prostate cancer, chronic myeloid leukemia, breast cancer, epilepsy	^35–37, 39–41, 61^
*GSTT1*	GSTT1*O	Gene deletion	600436	Uterine leiomyoma, hypertension, oral leukoplakia, brain tumour, breast cancer, coronary heart disease, psoriasis^a^, epilepsy	^35–38, 40,43, 44^
*GSTP1*	GSTP1*B	Ile105Val	134660.0002	chronic myeloid leukemia, Parkinson’s disease, Amyotrophic lateral sclerosis	^39,56, 66^
	GSTP1*C	Ile105Val/Ala114Val	134660.0003	Alzheimer’s disease, Parkinson’s disease	^52, 56^
	GSTP1*D	Ala114Val	—	Brain tumour, Parkinson’s disease	^38, 56^
*GSTA1*	GSTA1*B	-69(C/T), mutation promoter	138359	Psoriasis^b^	^44^
*GSTO1*	GSTO1*B	Glu155 deletion	605482	Alzheimer’s disease	^53^
*GSTO2*	GSTO2*B ^c^	Asn142Asp	612314	Breast cancer	^42^
	GSTO2*C ^c^	Ala183Gly	—	Spinocerebellar ataxia type 2	^65^

*OMIM* Online Mendelian Inheritance in Man

^a^ in association with GSTA1*A

^b^ in association with GSTT1*A

^c^ Allelic variants were identified as B and C in this work following the nomenclature by Townsend and Tew^34^

Notably, GST variants have the ability to influence response to drugs and environmental stresses^32,45^. For instance, it has been recently observed that in prostate cancer patients, polymorphism in GSTM3-3 may contribute to resistance to hormonal therapy through oxidative stress^45^. Indeed, in several studies it has been proposed that GST polymorphisms may be used as biomarkers for prognosis in cancer patients^39,45^.

### Neurodegenerative diseases

GST polymorphisms have also been associated with neurodegenerative conditions including Alzhaimer and Parkinson diseases (Table 1)^46,47^. Oxidative stress is one of the events that possibly contributes to the development and progression of neurodegenerative disorders^48,49^. Oxidative stress is caused by an overproduction of oxidants or unbalanced defence mechanisms played by antioxidants and their related enzymes. One example is the dysregulation of GSH homeostasis and alterated levels or functions of GSTs (both decrease or increase)^48,49^. In the nervous system, the role exerted by GSH and its related enzymes is particularly relevant since neurons are highly sensitive to oxidative stress^50^.

#### Alzhaimer’s disease

Alzhaimer’s disease (AD) is a chronic neurodegenerative disease characterized by the pathological accumulation of β-amyloid peptides and neurofibrillary tangles into the brain, that usually starts slowly and gets worse over time up to dementia. Several studies have analyzed GST status in AD patients. In particular, reduced GST activity as compared to control subjects was described in all brain areas and ventricular CSF in postmortem AD patients, suggesting a role of these enzymes in the pathogenesis of the disease^51^. A significant presence of a GSTP1-1 allelic variant (the GSTP1*C allelic variant) was found in late-onset AD patients^52^. It has been proposed that the allelic variant might influence the stability of GSTP1-1/JNK complex and consequently JNK activity, depending on the redox status of the cell. Recent results also suggested that an uncommon polymorphic variant of GSTO1-1—the GSTO1*E155del variant—was associated with AD risk. It was shown that the deletion of E155 in GSTO1-1 has a significant effect on enzymatic stability and may consequently alter the physiologic role of the enzyme in the brain regions^53^.

#### Parkinson’s disease

Parkinson’s disease (PD) is a long-term neurodegenerative disorder that affects the motor system, characterized primarily by the death of dopaminergic neurons in the substantia nigra and the presence of intracellular aggregates of misfolded α-synucleine (Lewy bodies) in the surviving neurons^54,55^. In addition to movement disorder, different non-motor symptoms such as dementia, psychosis and depression are observed in patients, which increase in late stages of disease. A correlation between severe reduction of GSH levels and PD onset was recently proposed^50^. Furthermore, several studies were conducted on various classes and isoforms of GSTs to obtain informations about their association with increased risk for the development of the disease^47,50^. In a proteomic study on postmortem samples of PD patients it was shown that GSTP1-1 levels are increased in PD patients at advanced stages. A role of the enzyme in modulating stress responses by controlling JNK activity was also proposed^56, 57^. Studies on GSTO1-1 also suggested a protective effect of this enzyme on PD, exerted through the inhibition of the active form of interleukin-1β (IL-1 β) which is a critical factor in inflammatory response^47,58^.

#### Epilepsy

Epilepsy is a disease of the brain, the distinctive characteristic of which is a predisposition to generate unprovoked seizures^59^. The association between polymorphisms of GST genes and the risk of epilepsy has been investigated in several studies^60–62^. In patients with progressive myoclonus epilepsy, a *GSTT1*-null genotype has been associated with increased risk of developing the disease where it might contribute to enhanced susceptibility to oxidative stress^60^. Different results were obtained in other group of patients^61^. In this study both *GSTM1* and *GSTT1* null genotypes were analysed to evaluate the effects on epilepsy risk susceptibility. The results obtained revealed that *GSTM1* null genotype and not *GSTT1* null genotype is significantly associated with epilepsy and possibly involved in the development of the disease. GSTs are also involved in antiepileptic drug resistance. It has also been observed a correlation between high levels of GSTP1-1 in the brain and medical intractability, suggesting that the enzyme may contribute to resistance to antiepileptic drug treatment^62^.

#### Spinocerebellar ataxia type 2

Spinocerebellar ataxia type 2—a genetic neurodegenerative disorder due to a CAG repeat expansion mutation in the *ATXN2* gene coding for the protein ataxin-2^63^—has been associated to oxidative stress, in particular due to alterations in the enzymatic activity of antioxidant enzymes including GSTs^64^. In a case-control study, it was observed a significant increase in GST activity in affected individuals relative to controls. More recently, spinocerebellar ataxia type 2 has also been linked to the presence of a transition polymorphism in the *GSTO2* gene (rs2297235 “A183G”) which significantly correlates with the age at disease onset; the presence of at least one G allele causing an anticipated disease onset of 5.4 years^65^.

#### Amyotrophic lateral sclerosis

Amyotrophic lateral sclerosis (ALS) is a fatal progressive neurological disorder characterized by muscle paralysis caused by the degeneration of motor neurons in the brain, brainstem and spinal cord. High levels of oxidative stress may contribute to ALS onset, increasing motoneurons death, and heavy metals may be a cause for the increase of ROS. Under this frame, it was observed that the association between lead exposure and ALS risk may correlate with particular *GSTP1* gene polymorphisms^66^. Indeed, the expression of the GSTP1-1 variant Ile105Val is able to increase the effect of lead on the development of ASL^66^. Finally, a possible effect of the *GSTO1* and *GSTO2* loci on the age at onset of ALS was also reported^67^.

## ROLE of GSTs in cancer drug resistance

Multidrug resistance is an event involving several different mechanisms^68^. In a broad variety of cancers, GSTs are involved in the resistance to several anticancer drugs by their conjugating activity (some examples are reported below). In cancer cells, GSTs often show high levels of expression when compared to normal cells^1,69^. Overexpression of GSTs may contribute to increase detoxification of anticancer drugs^68^. Synergistic interactions between GSTs and efflux pumps have also been observed in several studies^68,70^. Indeed, drug resistance is also related to increase discharge (efflux) of anticancer drugs due to the overexpression of the efflux transporters. GS-conjugates are actively transported out of cells by efflux transporters including MRP1 and P-glycoprotein belonging to the superfamily of ATP-binding cassette transporters^70–72^. Therefore both GST and efflux pumps overexpression may confer high levels of resistance to the cytotoxic action of several antineoplastic drugs. In addition, GSTs are also involved in conferring multidrug resistance with a non-catalytic mechanism through their inhibition of the JNK signaling pathway, an event that protects tumoral cells from apoptosis, as it will be detailed later.

A few examples of the activities played by GSTs on antineoplastic agents and their contribution to drug resistance in cancer are the following:

### Cisplatin

Platinum chemotherapeutic drugs are among the most extensively used anticancer agents^73^. Cisplatin is one of the most efficacious, and, although its use produces several side-effects, it is still a drug of choise for treating a number of solid cancer^74,75^. It interacts with DNA to form adducts—such as DNA–DNA and DNA–proteins crosslinks—which activate several signal transduction pathways that trigger cell death^74,76^. Several and unrelated mechanisms are responsible for drug resistance to cisplatin. One of them is the ability of GSH to bind and inactivate the anti-cancer drug^76^. High concentrations of the thiol were detected in the presence of cisplatin, decreasing the levels of the available drug. Although the conjugation of GSH to cisplatin also happens non-enzymatically, GSTs can catalyse it (Fig. 3d). It has been observed that enzymatic inactivation of cisplatin by GSTP1-1 contributes significantly to drug-resistance^77^.

### Busulfan

Busulfan is an alkylating agent widely used in myeloablative conditioning regimens before bone marrow or hematopoietic stem cell transplantations^78^. High-dose busulfan treatment is correlated with drug-related events such as cataracts and hepatic sinusoidal obstruction syndrome. The toxicity of busulfan is caused by its irreversibile glutathionylation played by GSTs. GSTs, primarily GSTA1-1, are involved in the first step of the metabolic pathway of busulfan^79,80^. GSTs catalyse busulfan conjugation to GSH, yielding glutathionyl-tetrahydrothiophene (GS-THT^+^; Fig. 3e). Then, GS-THT^+^ is metabolized via a β-elimination reaction to obtain γ-glutamyl-dehydroalanyl-glycine (EdAG), which reacts with a second GSH to form lanthionine GSG, a non-reducible analogue of GSH. Lanthionine GSG in turn, forms an irreversible mixed disulfide with protein thiols that could lead to dysregulation of proteins that are normally regulated by reversible glutathionylation^81^.

### Dichloroacetate

Dichloroacetate (DCA), a product of water chlorination, is considered for the treatment of several disorders including genetic mitochondrial diseases and some hyperproliferative conditions^82^. DCA inhibits mitochondrial pyruvate dehydrogenase kinase that inactives the pyruvate dehydrogenase complex. The maintenance in the active state of pyruvate dehydrogenase complex – that oxidizes pyruvate to acetyl CoA – stimulates oxidative phosphorylation. In tumor cells, a switch of glucose metabolism from aerobic glycolysis to oxidation leads to inhibition of proliferation and induction of caspase-mediated apoptosis^82^. A GST of the zeta class (GSTZ1-1) is involved in the metabolism of dichloroacetate^83,84^. GSTZ1-1 is a bifunctional enzyme that, acting as maleylacetoacetate isomerase, is also involved in the metabolic degradation of phenylalanine and tyrosine^83^. The enzyme dechlorinates DCA to glyoxylate inactivating it (Fig. 3f) and subsequently may provide resistance to DCA treatment. Intriguingly, it has also been observed that DCA is able to inhibit GSTZ1-1^85^. It has been observed that repeated doses of DCA resulted in loss of GSTZ1-1 protein and activity, probably due to post-translationally modifications of the enzyme^85^. Recently, it has also been demonstrated that abnormal regulation of GSTZ1-1 expression in cancer cells may influence DCA metabolism, and consequently lead to an altered therapeutic response^84^.

## GST inhibitors

Inhibitors of GSTs may increase the sensitivity of cancer cells to antitumor drugs and thus they may be used for several therapeutic applications^86^. For this reason, a remarkable number of inhibitors for GSTs have been synthesized as well as GSH analogues with better specificities and reduced toxicities^69,87^. Furthermore, different natural inhibitors found in plants were also discovered and investigated^24,88^. Here we describe some of the most characterized examples:

### Ethacrynic acid and analogues

**Fig. 5:**
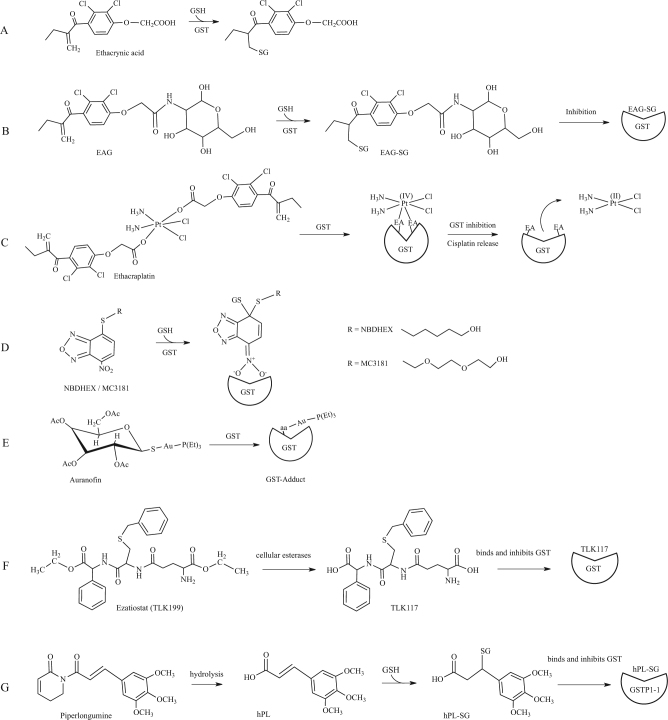
GST inhibitors. **a** GST-catalyzed conjugation of ethacrynic acid; **b** A conjugate of ethacrynic acid and glucosamine (EAG) reacts with GSH and inhibits GSTs; **c** Ethacraplatin is a Pt(IV)-complex compound which contains two ethacrynic acid moieties. When exposed to GSTs, this compound inhibits the enzyme and liberates cisplatin; **d** NBDHEX and its MC3181 derivative are GST inhibitors that bind the enzyme H-site and are conjugated by GSH leading to the formation of a stable σ complex; **e** Auranofin structure and its inhibitory effect on GSTs; **f** Ezatiostat (TLK199) is a GSH analogue that exerts its inhibitory effects on GSTs through G-site binding; **g** Piperlongumine is hydrolized inside cells and forms a conjugate with GSH that exerts inhibition of GST acitivity

Ethacrynic acid (EA)—an α,β-unsaturated ketone used as diuretic drug—is a common substrate/inhibitor of several GSTs (Fig. 5a)^68,89^. EA is active against human tumor cells in particular through its inhibitory activity on GSTP1-1 by covalent binding of the GSH-EA complex. However, despite the good pharmacological properties shown by this molecule on several cancers, its use in the clinic is problematic because of its strong diuretic properties. For this reasons, several laboratories have developed EA analogues with improved properties. For example, EA was conjugated to 2-amino-2-deoxy-d-glucose (EAG)^90^, obtaining an adduct with significant anticancer activity such as EA but without the undesired diuretic activity^90^. EAG is structurally similar to EA, is conjugated by GSH and the adduct inhibits GSTP1-1 (Fig. 5b). More recently, the anticancer activity of new EA derivates has been tested in antiproliferative assays on two different tumoral cell lines^91^. These new molecules showed an efficient anti-proliferative activities against human cancer cells emphasizing the potential of them as novel anticancer agents.

### Ethacraplatin

Ethacraplatin is a bifunctional drug developed to overcome GST mediated cisplatin resistance^92^. Ethacraplatin – a dual-threat platinum (IV) prodrug – is a cisplatin molecule coordinated to two ethacrynic acid ligands which is able to inhibit irreversibly GSTP1-1 enzymatic activity being reduced and cleaved as a consequence of binding (Fig. 5c)^73,93^. This in turn permits to increase the diffusion of Pt ions and reverts platinum drug resistance^93^. Ethacraplatin is also able to revert cisplatin resistance in microsomal GST1-1 overexpressing cells^94^. Recently, a new potential drug preparation was developed encapsulating ethacraplatin in nanoscale micelles. This significantly enhanced the accumulation of cisplatin in tumor cells, increasing efficacy in cisplatin resistant cells and decreased tossicity^95^.

### NBDHEX

6-(7-nitro-2,1,3-benzoxadiazol-4-ylthio)hexanol (NBDHEX) is a highly efficient inhibitor of GSTP1-1 and other GSTs, which triggers apoptosis in several cancer cells (Fig. 4)^96,97^. As already mentioned, GSTP1-1 is over-expressed in several cancers where it protects cells from cell death by inhibiting the activity of JNK or its upstream activation. Indeed, the formation of both GSTP1-JNK and GSTP1-1-TRAF2 complexes were described in vivo. NBDHEX binds the GSTP1-1 H-site and forms a complex with GSH, inactivating the enzyme (Figs. 4 and 5d)^98^. Importantly, NBDHEX is also able to dissociate GSTP1-1 from its complexes with both JNK and TRAF2, thus enabling their activation^99^. Drug combination studies showed that NBDHEX is significantly active on cisplatin-resistant human osteosarcoma cells^77^.

Oner of the problems in the clinical use of NBDHEX may arise from its lack of specificity for GSTP1-1. For example this drug displays a much higher affinity for GSTM2-2 than for GSTP1-1^98^. For this reason, several novel NBDHEX analogues, with improved selectivity for GSTP1-1, have been synthesized and characterized to obtain new therapeutic opportunities for the treatment of drug resistant tumors including human melanoma^100,101^. Among them, the MC3181 derivative was recently found to be extremely efficient in blocking cancer growth and metastasis in a xenograft mouse model of vemurafenib resistant melanoma (Fig. 5d)^102^.

### Auranofin

Auranofin is an antiarthritic gold phosphine compound that also exhibits anticancer effects similar to those of cisplatin. Unlike cisplatin and similarly to other antiarthritic gold drugs, auranofin appears to exert its activity through inhibition of enzyme activities rather than DNA damage. This different molecular mechanism would allow to overcome cell resistance against platinum agents. Recent studies suggested that one of the enzymes inhibited by auranofin is GSTP1-1 (Fig. 5e)^103^. Notably, similar inhibitions were observed both with the wild type enzyme and its cysteine mutants, suggesting that thiol conjugation is dispensable for GST inactivation by auronofin in contrast to other described targets^103^. Therefore, future research efforts will be necessary to clarify the mechanism of enzyme inhibition played by auranofin on GSTs. Finally, it is of notable interest that the well-described antiplasmodial effects of auranofin may be possibly correlated to GST inhibition.

### Glutathione analogues

Different GST inhibitors have been conceived based on the structure of the GSH moiety. The tripeptide GSH is the most abundant low molecular weight thiol in the cell, and it is found is almost all eukaryotes and several prokaryotes^104^. It has been observed that the increase of GSH levels has a cytoprotective effect. Nevertheless, since GSH cannot be administered directly—because of its insufficient absorption, stability and solubility—analogues have been obtained, through modifications at the γ-glutamyl, cysteine and glycine moieties, and/or the functionalisation of the central cysteine, that are able to inhibit the activity of GSTs^69,87,105^. A well-characterized example is ezatiostat (TLK199)—a GSH peptidomimetic molecule—designed to display enhanced cellular uptake and whose metabolites bind the G-site of GSTP1-1 causing enzyme inhibition. Among them, TLK117 results from de-esterification of TLK199 and is the most selective GSTP1-1 inhibitor (Fig. 5f). Its interaction with the enzyme results in JNK activation and c-Jun phophorilation^106^. This compound was found efficaceous in the treatment of myelodisplastic syndrome and its investigation has so far moved up to phase I-IIa clinical trials. Moreover, it has been observed that cyclic GSH, which is less sensitive to the effects of several degradative enzymes, appears to have toxic activity on cancer cell lines^87^.

### Piperlongumine

Piperlongumine (PL) is an alkaloid isolated from *Piper* species, commonly used in traditional medicine – in particular in Asia and Latin America – that in recent years has been extensively studied because of its anti-cancer potential, being able to inhibit proliferation in several cancer cell lines^107^. It has been observed that the anti-cancer effects are associated with an increase of reactive oxygen species (ROS) and reduction of GSH levels. Recently, it has been demonstrated that PL inhibits GSTP1-1 by directly binding the enzyme^88^. A structural model of the interaction suggests that the active molecule is a hydrolyzed form of PL (hPL) which forms a complex with GSH in the enzyme active site. Authors proposed a model wherein PL enters the cell, is hydrolyzed in its active form (hPL) with subsequent formation of a hPL:GSH complex that binds the active site of GSTP1-1 causing its inhibition (Fig. 5g). The following reduction of GSH cell concentration and increase of ROS levels also contributes to cell death^88^.

## Pro-drugs

Prodrugs are pharmacologically inactive molecules in vitro that are converted into their active parent drugs in vivo after chemical modifications and/or enzymatic reactions. They are often designed to improve the bioavailability of active drugs by increasing their amount in target cells and reducing off-target effects. The overexpression of GSTs in cancer cells offers unique opportunities for prodrug therapy^108^. Indeed the activation of prodrugs in GST overexpressing cells may lead to high concentrations of an active drug, as compared to normal cells with moderate enzyme levels. Essentially, two groups can be distinguished: (i) molecules containing GSH or GSH-like moiety and (ii) molecules whose enzymatic activation occurs through a GSH-conjugate intermediate^109^. Important examples are detailed below.

### Canfosfamide

**Fig. 6:**
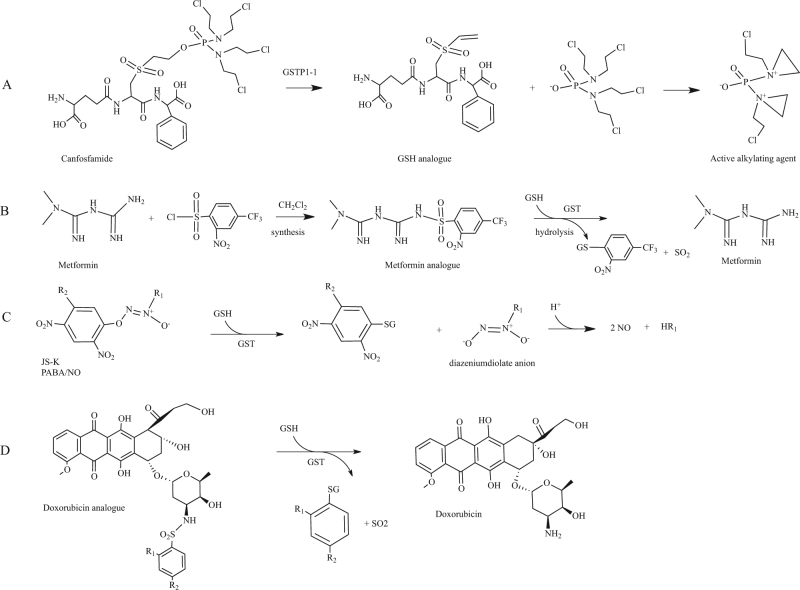
GST prodrugs. **a** Bioconversion of canfosfamide in an active alkilating metabolite played by GSTP1-1; **b** synthesis and bioconversion played by GST of a metformin sulfonamide prodrug; **c** GSH conjugation with nitric oxide releasing agents to form diazeniumdiolate anion and subsequent release of two NO molecules; **d** Release of active doxorubicin by GST sulfomidase activity played on nitrobenzenesulfonyl analogues

Canfosfamide, also known as TLK286, is a GSH analogue activated by GSTP1-1 into a GSH derivative and phosphorodiaminate, an alkylating metabolite that forms covalent linkages with DNA (Fig. 6a)^110,111^. TLK286 clinical effects were investigated in the last decade in phase II and phase III trials for the treatment of drug-resistant ovarian cancer^112,113^. A phase I-IIa clinical trial in combination with carboplatin and paclitaxel investigated the effect of canfosfamide in advanced non-small cell lung cancer^114^.

### Metformin analogues

Metformin is the most used drug in the treatment of type II diabetes in the world. The drug reduces hepatic glucose production, enhances glucose uptake in the peripheral tissues and increases insulin sensitivity^115^. Indeed metformin inhibits the mitochondrial oxidative phosphorylation complex I, leading to increased cellular AMP levels and subsequent stimulation of AMPK, an enzyme that plays key roles in the regulation of cellular energy homeostatis. However, metformin also exerts anticancer effects and is thought to do this through both AMPK-dependent and AMPK-independent mechanisms^116^. Several studies showed evident association between type II diabetes and cancer, in particular in liver and pancreas, organs persistently exposed to high levels of endogenous insulin^117–119^. Although metformin is extremely efficient, its absorbtion is poor and variable. On the other hands, great amounts of drug cause gastrointestinal distress that is not always well tolerated by patients. To enhance the oral absorbtion of the drug, metformin prodrugs were developed with improved permeability across the lipid membrane^120^. The first developed metformin prodrug, namely its cyclohexyl sulfenamide derivative, was in part activated on the apical side of the enterocytes with consequent decrease of absorbtion^121^. To overcome the problem, prodrugs activated only after oral absorption were developed. Since high levels of GSTs are expressed in the hepatocytes and it has previously been demonstrated that GSTs display sulfonamidase activity, three sulfonamide prodrugs of metformin were designed and synthesized^120^. GSTs catalyze the GSH-mediated hydrolysis of sulphonamide bonds to form the corresponding amine^108^. For one of these derivatives, authors obtained interesting results. GST was able to convert the derivative into the parent drug in a quantitative manner (Fig. 6b).

### Nitric oxide prodrugs

Nitric oxide (NO) is involved in several key physiological and pathological processes^122^. In cancer cells, high concentration of NO can damage various macromolecules such as nucleic acids and proteins and finally trigger apoptosis. NO-releasing agents, such as JS-K (*O*^2^-(2,4-dinitrophenyl)-1-[(4-ethoxycarbonyl)piperazin-1-yl]diazen-1-ium-1,2-diolate)^123^ and PABA/NO (*O*^2^-[2,4-dinitro-5-(*N*-methyl-*N*-4 carboxyphenylamino)phenyl] 1-*N*,*N*-dimethylamino)diazen-1-ium-1,2-diolate)^124^, are able to induce differentiation and cell death in a variety of cancer cells through GSH consumption, DNA synthesis inhibition and inhibition of enzymes involved in the defense against cell damage. GSTs catalyse the reaction of JS-K or PABA/NO with GSH to form a diazeniumdiolate anion that spontaneously decomposes, producing two equivalents of NO (Fig. 6c). To improve drug properties—such as stability and anticancer activity—of diazeniumdiolate-based NO donors, a number of new molecules were synthesized and biologicaly evaluated^109,125,126^.

### Doxorubicin analogues

Doxorubicin (DOX) is a common topoisomerase II inhibitor widely used in the treatment of solid tumors and malignant hematologic diseases. DOX is a highly toxic molecule and induces drug resistance due to the overexpression of ABCB1 efflux pump and other related proteins^127^. Microsomal GST1 (MGST1) and GSTP1-1, which are frequently overexpressed in cancer cells, contribute to DOX resistance. To overcome drug resistance and enhance its cytotoxicity, two derivatives of DOX were synthesized incorporating a sulfonamide moiety: 4-mononitrobenzenesulfonyl DOX (MNS-DOX) and 2,4-dinitrobenzenesulfonyl DOX (DNS-DOX)^94^. As reported above, GSTs display sulfonamidase activity. MNS-DOX and DNS-DOX were converted by MGST1 and GSTP1-1 via sulfonamide cleavage into the active parent drug (Fig. 6d). Notably, the less reactive MNS-DOX was more effective in MGST1 overexpressing cells than DNS-DOX. A new derivate of DOX, 4-acetyl-2-nitro-benzenesulfonyl doxorubicin (ANS-DOX) was synthesized and shown to be converted into DOX by MGST1 and GSTA1-1, whereas no activity was observed for GSTP1-1^128^. ANS-DOX showed to be toxic in GSTA1-1 overexpressing cancer cells. Collectively, these investigations suggested the plausibility of modulating the rate of drug release as well as specifically targeting different GSTs by modifying the sulfonamide moiety^128^.

## Conclusions

Recent advances in this field have highlighted the importance of GST activities and the role that these enzymes play in diverse cellular processes as well as in conferring resistance to chemotherapy. Within this context, it is not surprising that a great deal of inhibitors and pro-drugs targeting GSTs have been synthesized and tested so far, with new scaffolds or analogues being reported every year. Some of these molecules have also entered clinical trials and we will possibly welcome in the future the approval of a GST inhibitor or pro-drug for the treatment of patients. While surveying the literature in preparation of this manuscript we have also noticed and reported an increasing interest in the role that GSTs play in neurodegeneration, where isozymes belonging to the family may be either up-regulated, mutated or absent, according to the different diseases. Research in this field is particularly lively and holds the promise to define specific GSTs, or specific GST polymorphic forms as possible pharmacological targets.

Up to know, the majority of established and newly synthesized GST inhibitors have been primarily tested for their cytotoxic effect in cancer cell lines and mouse xenograft models, showing promising activities. One of the problems that have emerged, especially when many of these drugs have been tested in vitro for their inhibition properties, is their lack of specificity. Indeed, while in many cases it would be desirable or crucial to specifically target a particular GST isozyme or polymorphic form, this is hardly achievable with the molecules synthesized and investigated so far. We anticipate that the search for more specific GST inhibitors will be the main goal of future research in this field.
